# Papillomavirus is not detected in benign neoplasms of the canine Meibomian gland despite evidence of HPV-mediated tumorigenesis in the human Meibomian gland

**DOI:** 10.3389/fvets.2026.1812954

**Published:** 2026-04-09

**Authors:** Victoria Rushtlion, Isabella Boyack, Autumn Berlied, Cornelia Peterson

**Affiliations:** 1 Department of Comparative Pathobiology, Cummings School of Veterinary Medicine, Tufts University, North Grafton, MA, United States; 2 Tufts Center for Vision Research, Boston, MA, United States

**Keywords:** adenoma, canine, epithelioma, formalin-fixed paraffin-embedded tissue, polymerase chain reaction, sebaceous carcinoma, viral papilloma

## Abstract

**Objective:**

Meibomian gland (MG) tumors are common in dogs. Papillomaviruses have been detected in epithelial neoplasms across species, including those arising from the human MG, but their role in the development of canine MG tumors has not been investigated. The purpose of this study was to evaluate whether Canine Papillomavirus (CPV) contributes to MG tumorigenesis.

**Methods:**

A retrospective review of a Laboratory Information Management System (LIMS) was performed to identify cases of canine MG tumors submitted between 2019 and 2024. DNA was extracted from archival FFPE tissue scrolls, and PCR was performed using validated viral CPV-1 E6 and L1 primers. Clinicopathologic features including patient age and sex, tumor size, tumor laterality and location, presence of chalazion, mitotic count, and extent of surgical excision were evaluated.

**Results:**

LIMS review yielded 106 cases of canine MG tumors, and 102 were histologically confirmed to be MG adenomas or epitheliomas. PCR was performed on 99 samples, and no viral amplicons were detected in any MG tumor. Dogs with adenomas were younger than those with epitheliomas. There were no differences in sex or alteration status between tumor groups, and Labrador retrievers and Poodles were overrepresented. There were no differences between tumor types in terms of laterality or upper vs. lower eyelid. Adenomas were smaller and had lower mitotic counts compared to epitheliomas. The presence of chalazion or completeness of the surgical excision was not different between tumor subtypes.

**Conclusion:**

While we confirm consistent morphologic and phenotypic differences between canine MG adenomas and epitheliomas, these results suggest that, unlike their human counterparts, neither tumor subtype is virally mediated.

## Introduction

Benign adenomas and epitheliomas of the Meibomian glands (MGs) are the most frequently diagnosed eyelid margin tumors in middle-aged to older dogs (1, 2). Both adenomas and epitheliomas arise from the MGs, which are specialized sebaceous glands embedded within the upper and lower eyelids and play a key role in secreting the lipid component of the tear film (3, 4). While often regarded primarily as a cosmetic concern, MG tumors may lead to complications through contact with the corneal surface, often resulting in mechanical irritation and ulceration (5). Histologically, the distinction between adenomas and epitheliomas is based on the relative proportion of basal cells to mature meibocytes. Epitheliomas exhibit a higher proportion of sheet-like basaloid cells, giving them a more undifferentiated appearance, whereas adenomas consist predominantly of mature, well-differentiated meibocytes characterized by a more foamy, lipid-filled cytoplasm (3).

Ocular adnexal sebaceous carcinoma (SebCA) is a rare but aggressive human eyelid malignancy, most often arising from the MG, characterized by multifocal intraepithelial and infiltrative growth which often necessitates disfiguring surgery such as orbital exenteration to manage local control (6). OA SebCA is the second most common eyelid malignancy in humans, occurring most frequently in older women and individuals of Asian descent (5). These tumors are often misdiagnosed as unilateral blepharoconjunctivitis or recurrent chalazion due to their nonspecific clinical presentation (5). However, they are histologically aggressive neoplasms characterized by local invasion as well as potential for both lymphatic and hematogenous metastasis (5).

A subset of ocular adnexal SebCA has been linked to Human Papillomavirus (HPV), indicating a possible viral contribution to pathogenesis and the presence of biologically distinct subtypes within ocular adnexal SebCA (6, 7). Critically, both low and high-risk HPV have been detected in benign sebaceous neoplasms of the ocular adnexa including sebaceous adenomas and sebaceomas, often without the cytopathic features which are classically suggestive of papillomaviral infection (e.g., koilocytes and cytoplasmic vacuolation) (8). Due to the imprecise nature of relying on these histopathologic features of epithelial neoplasms alone for the detection of papillomaviral infection across species, molecular evaluations are widely considered the gold standard (9–11).

Papillomaviruses are non-enveloped, double-stranded DNA viruses that are typically highly species-specific (12). While most infections are subclinical, certain viral types can alter host cell growth and differentiation through inhibition of tumor suppressors, leading to the development of papillomas, or less commonly, epithelial malignancies (i.e., carcinomas) (13, 14). Evidence from human and veterinary literature suggests an oncogenic role for papillomaviruses in epithelia, with viral DNA identified in vulvar carcinomas with sebaceous differentiation, equine cutaneous plaques, feline sebaceous tumors, and with respect to the ocular surface, canine conjunctival lesions (2, 3, 5, 15). Clinical diagnosis of papillomaviral infection in dogs relies on histopathologic evaluation of tumoral tissue to identify viral cytopathic effects, PCR for DNA detection in biopsies or swabs using pan-PV or Canine Papillomavirus (CPV)-specific primers, immunohistochemistry, and *in situ* hybridization (16, 17).

Although papillomavirus-associated tumors are well-documented in cutaneous and mucosal sites across veterinary species and in both ocular adnexal and extraocular sebaceous neoplasms of humans, their role in canine sebaceous neoplasms remains unexplored, particularly in those originating from the MGs. The objective of this work was to evaluate whether CPV-1 plays a role in the oncogenesis of canine MG tumors through retrospective histopathologic review and PCR detection of viral DNA in archived tumor samples.

## Materials and methods

### Sample population

Our Laboratory Information Management System (LIMS) was queried for cases containing the search terms: *canine*, *Meibomian gland tumor*, *epithelioma*, *adenoma*, and *adenocarcinoma* from 2019 to 2024. One hundred and six cases met these search criteria, and the hematoxylin and eosin (H&E)-stained slides were retrieved from the archives for diagnosis confirmation by a board-certified veterinary pathologist. Of these cases, 102 were confirmed to be benign MG adenomas or epitheliomas. Details regarding patient signalment, tumor clinical descriptions or gross findings, and histologic features were obtained from the corresponding electronic medical records and diagnostic pathology reports. Archived formalin-fixed, paraffin-embedded (FFPE) tissue blocks associated with these cases were retrieved.

### Polymerase chain reaction

Three 4 μm-thick scrolls were aseptically generated from each FFPE tissue block which had sufficient tissue remaining (*n* = 102), and total DNA was extracted using the QIAamp DNA FFPE Advanced Kit (Qiagen, Germantown, MD, United States). The concentration and 260/280 ratios were measured for each DNA sample using a Nanodrop spectrophotometer (ThermoFisher Scientific, Waltham, MA, United States), with 99 samples meeting quality thresholds (1.8 ≤ 260/280 ≤ 2.0; Supplementary Figure S1).

Polymerase chain reaction (PCR) optimization was performed using positive controls consisting of DNA extracted from FFPE sections of histologically diagnosed canine mucosal and cutaneous viral papillomas submitted to the diagnostic laboratory during the same period as the MG tumoral samples. Control DNA was amplified using primer sets targeting CPV-1 as shown in Table 1 (18).

**Table 1:** Description of primers utilized to examine canine Meibomian gland tumors for the presence of papillomavirus.

Primer	Polarity	Sequence	Expected amplicon length (bp)
CPV-1 L1	F	5′-CTT GTT TGG GGC TTA AGA GG-3′	261
	R	5′-TGC AGT GTG TAC CTG TCC TG-3′	
CPV-1 E6	F	5′-GGC ACT GTT ATC AAT GGA GC-3′	350
	R	5′-CAC ATA GTT CTT TGT CCG CC-3′	
Canine GAPDH	F	5′-TGA CAC CCA CTC TTC CAC CTT C-3′	191
	R	5′-CGG TTG CTG TAG CCA AAT TCA-3′	
BPV2 E5	F	5′-CAA AGG CAA GAC TTT CTG AAA CAT-3′	244
	R	5′-AGA CCT GTA CAG GAG CAC TCA A-3′	
FAP59	F	5′-TAA CWG T/ideoxyl/G G/ideoxyl/C AYC CWT ATT-3′	480
	R	5′-CCW ATA TCW VHC AT/ideoxyl/ TC/ideoxyl/ CCA TC-3′	

bp, base pairs; CPV-1, canine papillomavirus-1; GAPDH, glyceraldeyhyde-3-phosphate dehydrogenase; BPV2, bovine papillomavirus-2; FAP59, degenerate primer from human papillomavirus L1.

Due to the highly conserved sequences of papillomaviral oncogenes, additional validated primer sets targeting Bovine Papillomavirus-2 (BPV-2) E5 and FAP59 were also tested, as DNA amplification relies on these conserved regions rather than tissue-specific host DNA. However, viral amplification was not detected in control DNA using these primer sequences (18, 19). Two primer sets providing amplicons for CPV-1 (historically, canine oral papillomavirus; COPV) and one primer set providing an amplicon of a canine housekeeping gene (glyceraldehyde-3-phosphate dehydrogenase; GAPDH) were subsequently used to examine the MG tumors for the presence of viral DNA (18). Fifty nanograms of DNA in a total volume of 14 μL were utilized for each reaction, which was performed using an Eppendorf Mastercycler (Sigma-Aldrich, St. Louis, MO, United States) thermocycler and the following parameters: 5 min at 94 °C followed by 32 cycles of 60 s at 94 °C, 60 s at 58 °C, and 60 s at 72 °C before a 5 min incubation at 72 °C. PCR products were electrophoresed in 2% agarose gel and imaged using a LI-COR Odyssey Imaging System (LICORbio; Lincoln, NE, United States).

### Statistical analyses

Correlation of tumor clinicopathologic features, including patient sex/alteration status, tumor laterality and location, histologic presence of chalazion, and extent of surgical excision was analyzed by Fisher’s exact test with two-tailed *t*-tests. The distributions of patient age, greatest tumor dimension, and mitotic counts between adenomas and epitheliomas were assessed for normality using the Shapiro–Wilk test prior to evaluation by Student’s *t*-tests (patient age, greatest tumor dimension) or Mann–Whitney test (mitotic count), respectively. Spearman’s correlation tests were used to evaluate the relationship between greatest tumor dimension and mitotic count. All statistical analyses were performed using Prism 8 GraphPad (v. 10.5.0; San Diego, CA, United States) (α = 0.05).

## Results

**Figure 1:**
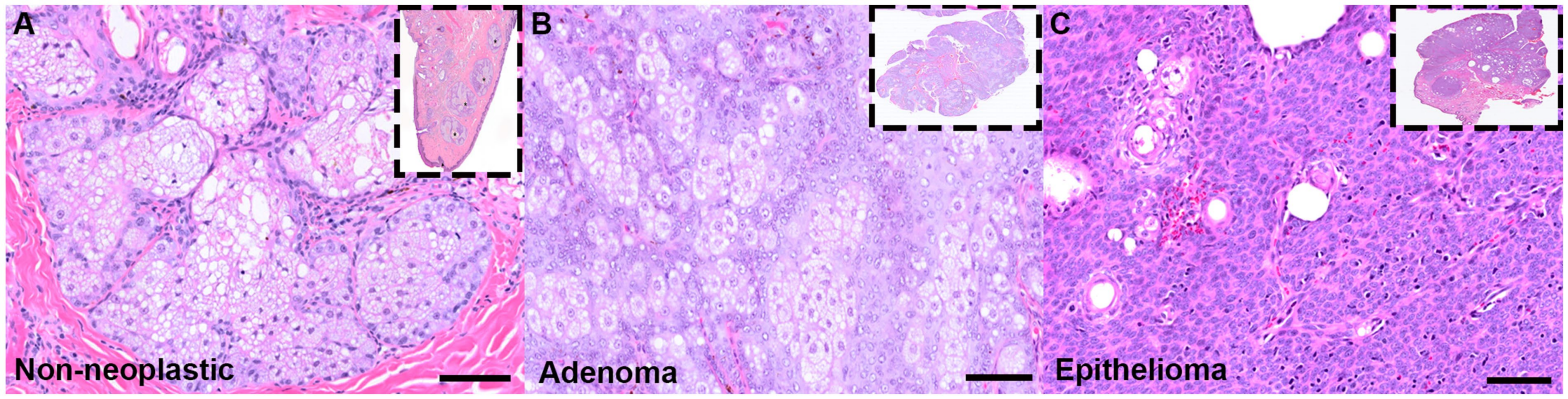
Representative photomicrographs of H&E-stained non-neoplastic and neoplastic canine Meibomian gland. **(A)** A non-neoplastic MG demonstrating acini of a holocrine gland surrounding a central duct. More central, differentiated meibocytes exhibit foamy, lipid-laden cytoplasm with less differentiated basaloid cells at the periphery. **(B)** A canine MG adenoma composed of haphazard lobules of predominantly of well-differentiated, lipid-laden meibocytes with foamy cytoplasm. **(C)** A canine MG epithelioma characterized by more sheet-like proliferations of undifferentiated basaloid cells with rarer sebaceous or squamous differentiation **(C)**. Scale bar: 50 μm (insets: 4X magnification of excised eyelid tissue).

A total of 106 cases were identified through LIMS review as canine MG tumors. Of these, 102 were histologically confirmed to be benign MG adenomas or epitheliomas (Figure 1). The additional cases were diagnosed as melanocytoma, hyperplasia, papilloma, and adenocarcinoma (*n* = 1 each). All corresponding FFPE tissue blocks were retrieved, although one was not utilized to generate a scroll due to insufficient tissue remaining embedded in paraffin. Following DNA extraction, three samples did not meet quality or quantity thresholds and were excluded from analysis. The remaining 99 samples were subjected to PCR amplification using primer sets targeting CPV-1 E6 and L1 regions and GAPDH.

**Figure 2:**
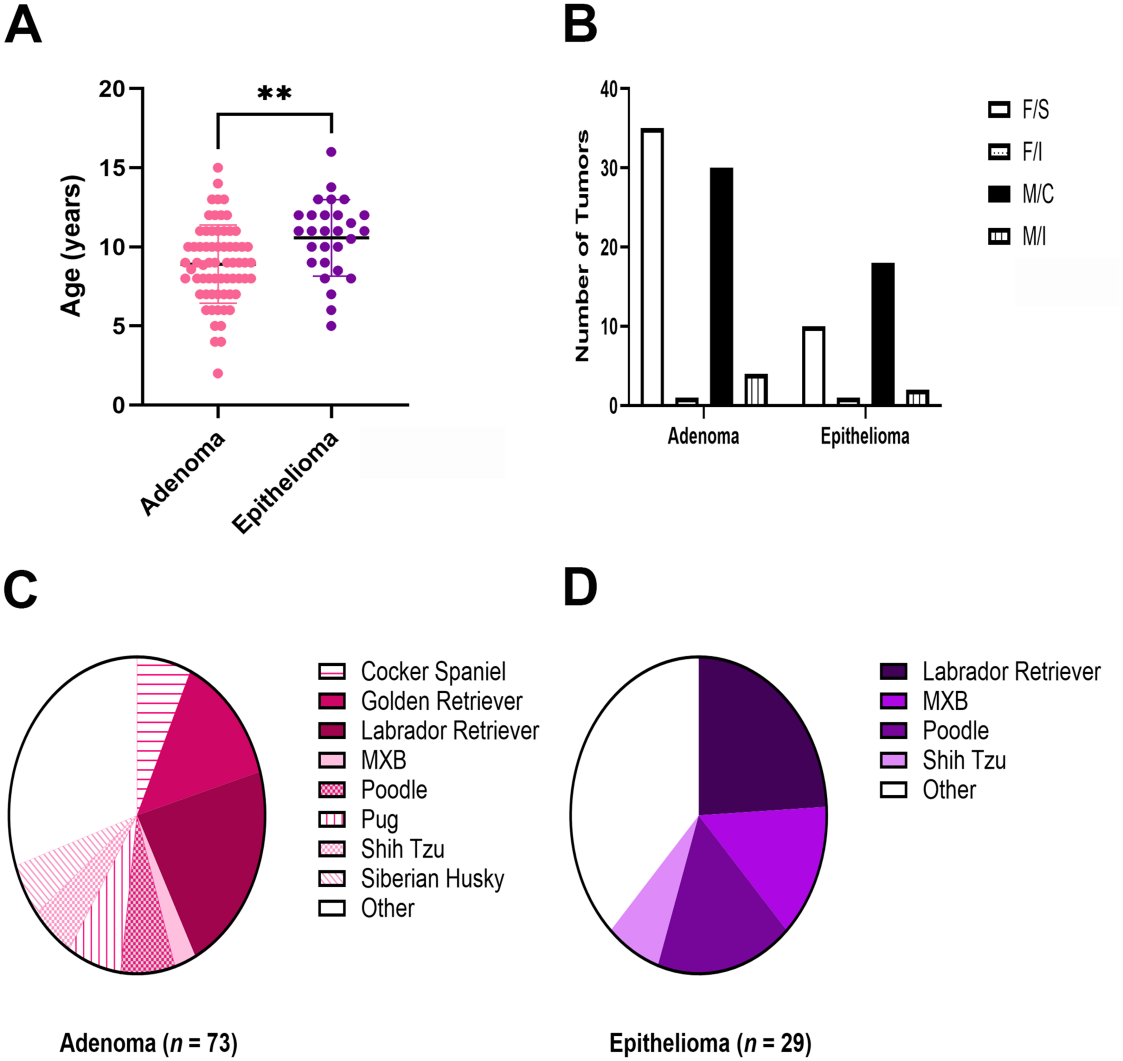
Comparison of signalment between canine Meibomian gland adenomas and epitheliomas including age **(A)**, sex and alteration status (**B**; F/S: spayed female; F/I: intact female; M/C: castrated male; M/I intact male), and breed **(C,D)**. **p < 0.01.

Dogs with adenomas were significantly (*p* = 0.0038) younger (8.92 ± 2.48 years) at the time of mass excision compared to those with epitheliomas (10.58 ± 2.42 years) (Figure 2A). There were no differences (*p* = 0.3095) in sex or alteration status between tumor types (Figure 2B). Overrepresented breeds with adenomas (Figure 2C) included: Labrador Retriever (*n* = 15), Golden Retriever (*n* = 10), Cocker Spaniel, Pug, and Poodle (*n* = 5 each). Labrador Retrievers (*n* = 7), Poodles (*n* = 5), and mixed breed dogs (MBD; *n* = 4) were overrepresented breeds in the epithelioma group (Figure 2D).

**Figure 3:**
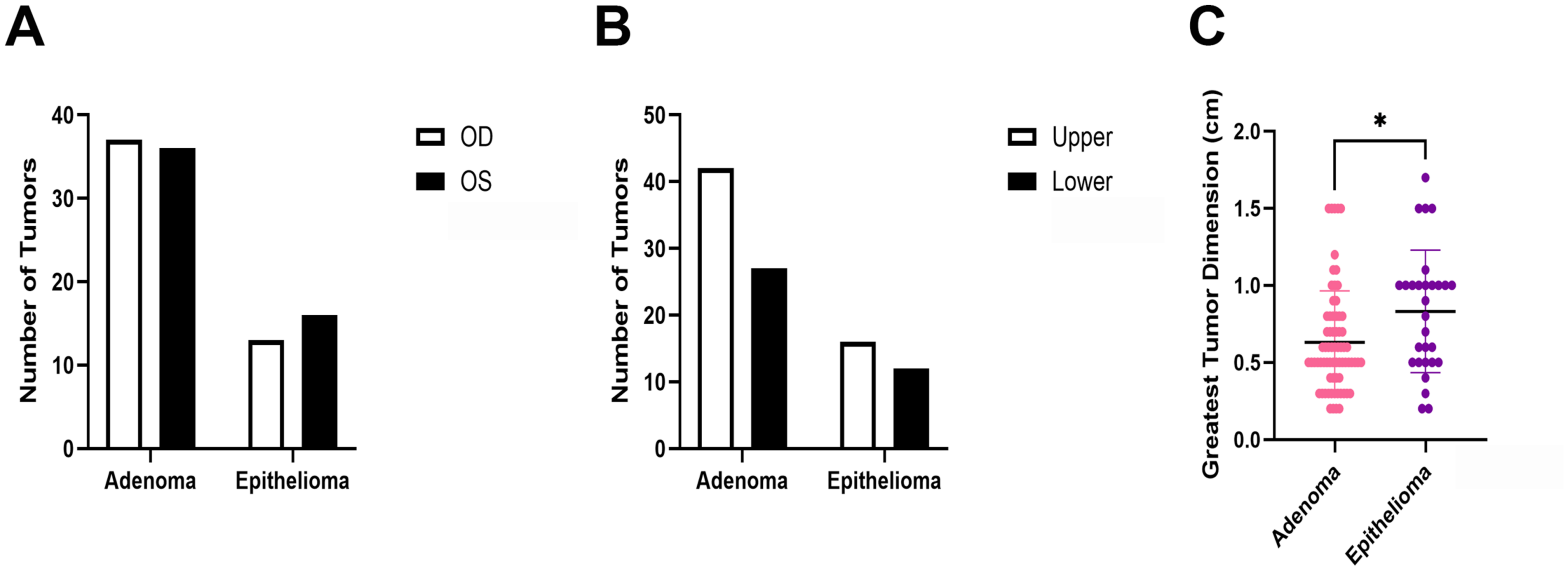
Comparison of gross features between canine Meibomian gland adenomas and epitheliomas including laterality (**A**; OD: oculus dexter, right eye; OS: oculus sinister, left eye), superior or inferior lid **(B)**, and greatest tumor dimension **(C)**. *p < 0.05.

There were no significant differences between tumor types in terms of laterality (OD: oculus dexter, right eye; OS: oculus sinister, left eye; *p* = 0.6635) (Figure 3A) or upper vs. lower eyelid (*p* = 0.8204) (Figure 3B). The mean greatest tumor dimension measured at grossing was significantly smaller (*p* = 0.0211) for adenomas (0.63 ± 0.3 cm) when compared to epitheliomas (0.83 ± 0.4 cm) (Figure 3C).

**Figure 4:**
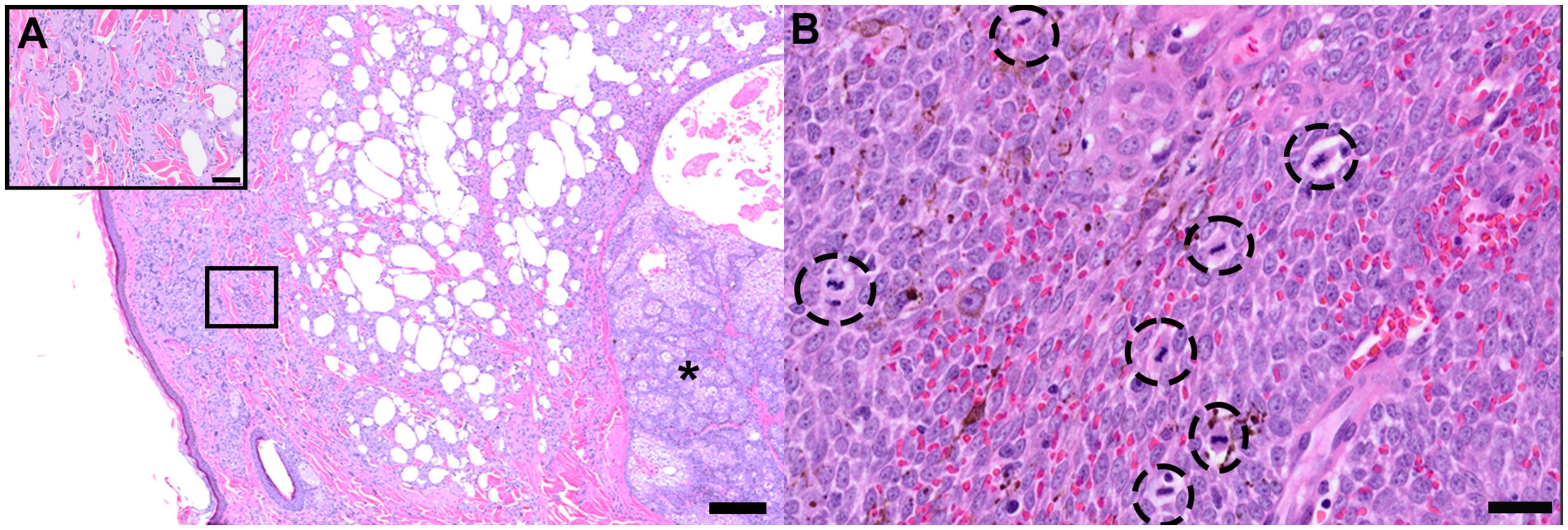
Histologic features of canine Meibomian gland tumors. Representative photomicrograph of an H&E-stained chalazion, typified by lymphohistiocytic to lipogranulomatous inflammation with multinucleated giant cells centered on pools of extracellular meibum adjacent the bulk of the tumor mass (asterisk). Scale bar: 200 μm. Inset: higher magnification of boxed region; scale bar: 50 μm **(A)**. Representative photomicrograph of an H&E-stained highly proliferative epithelioma with numerous mitotic figures (circled cells) Scale bar: 20 μm **(B)**.

**Figure 5:**
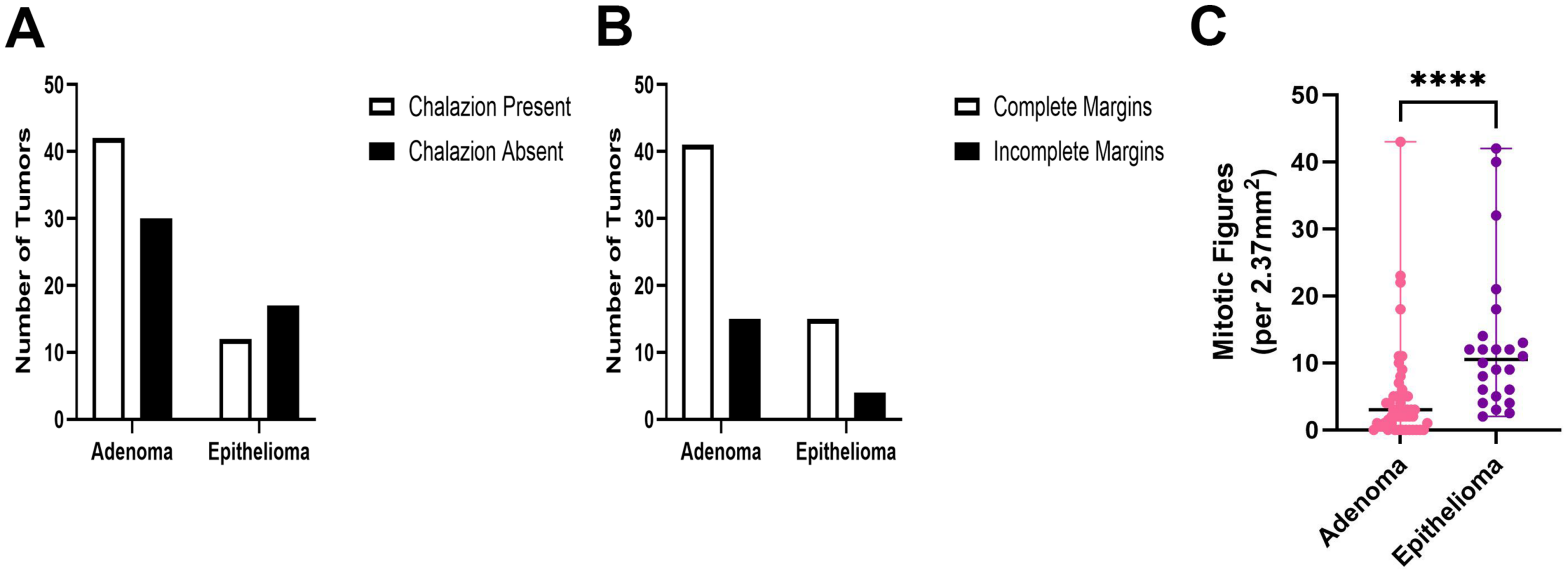
Comparison of histologic features between canine Meibomian gland adenomas and epitheliomas including presence of chalazion **(A)**, completeness of surgical excision **(B)**, and mitotic counts **(C)**. ****p ≤ 0.0001.

Both tumor subtypes demonstrated the features of chalazion, or the resultant lipogranulomatous inflammation centered on extracellular lipid (meibum) (Figure 4A), and both tumor subtypes exhibited robust mitotic activity (Figure 4B). There were no significant differences between tumor subtypes with respect to the presence of chalazion (*p* = 0.1308) (Figure 5A) or completeness of the surgical excision (*p* = 0.7647) as described in the corresponding pathology reports (Figure 5B). Adenomas demonstrated a significantly lower (*p* ≤ 0.0001) mitotic count (median: 3.0, range: 0–43 mitoses/2.37 mm^2^) than epitheliomas (median: 10.5, range: 2–42 mitoses/2.37 mm^2^) (Figure 5C). There were no significant correlations between greatest tumor dimension at grossing and mitotic count for adenomas (*R*^2^ = 0.006248, *p* = 0.5933) or epitheliomas (*R*^2^ = 0.03903, *p* = 0.3548; Supplementary Figure S2).

**Figure 6:**
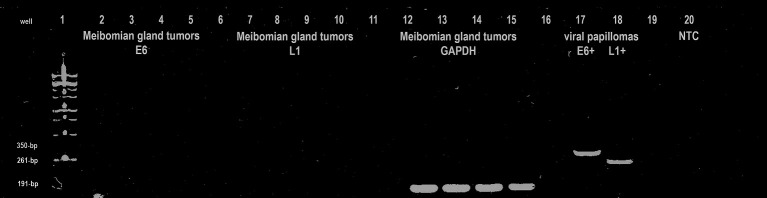
Representative gel of electrophoresed PCR product demonstrating validity of the utilized primer sets (E6: CPV-1 E6; L1: CPV-1 L1; GAPDH: glyceraldehyde-3-phosphate dehydrogenase, canine housekeeping gene; NTC: non-template control).

GAPDH amplicons (191 bp) were identified in all DNA samples obtained from MG tumors and viral papilloma controls (Figure 6). The CPV-1 E6 (350 bp) and L1 (261 bp) amplicons were only demonstrated by canine viral papillomas (positive controls), and no viral amplification was demonstrated in any canine MG tumor. No amplicons were observed in any non-template control (NTC) samples (negative controls).

## Discussion

Meibomian gland adenomas and epitheliomas are common benign eyelid tumors in dogs, yet their pathogenesis remains poorly defined. This study provides the first investigation into the possible link between papillomavirus infection and canine MG tumors. Canine Papillomavirus (CPV-1) was not detected in any of the DNA samples (*n* = 99) isolated from canine FFPE MG tumors (*n* = 102). These findings suggest that, unlike the a subset of human ocular surface and adnexal neoplasms in which papillomaviruses have been demonstrated in tissue section, papillomavirus does not appear to play a role in the oncogenesis of canine MG adenomas or epitheliomas (2–5). One of the key factors which may mediate these species-specific differences is papillomaviral tropism, with high-risk α-HPV serotypes exhibiting a strong tropism for the ocular surface and adnexal mucosa, and papillomaviruses of companion animals tending to favor the keratinizing squamous epithelia of the skin surface and oral cavity (20, 21). The potency of viral tumor suppression is high in HPV, with sustained E6 and E7 expression detected in sebaceous neoplasms of the human ocular surface and adnexa, while the papillomaviral oncogenes of the dog may require immunosuppression for malignant transformation (21, 22). Of all the potential mechanistic differences between human and canine papillomaviral infections, host factors are likely the most significant. Crucially, human papillomaviruses may be sexually-transmitted, while papillomaviruses with venereal transmission in veterinary medicine have been proposed only in select species including: swine, equine, bovine, ovine and cetacean (12, 23). Chronic ultraviolet radiation exposure and the pruritus-induced conjunctival epithelial microtrauma of atopy have also been correlated with HPV detection in human ocular surface and adnexal neoplasms, two additional factors with less relevance to shorter-lived veterinary species (9, 24–27).

The absence of CPV-1 DNA in the MG tumors evaluated here parallels the prior findings of Beckwith-Cohen et al. (28) and Schaefer et al. (19), respectively, who reported no viral involvement in benign squamous papillomas arising from the conjunctiva or orbital lobular adenomas. The absence of CPV-1 amplification cannot fully exclude prior or latent infection, or failure to detect other CPV types using the CPV-1 primers, particularly given the potential for either viral clearance or degradation of nucleic acids in FFPE tissue. However, DNA integrity was confirmed through amplification of a canine housekeeping gene, *GAPDH*, indicating that the lack of viral signal likely reflects true absence rather than inadequate sample quality.

Dogs in this cohort with adenomas were significantly younger than those with epitheliomas at the time of excision, which may suggest that adenomas represent an earlier stage or less biologically advanced lesion. Breed predispositions were noted in both tumor groups, with Labrador Retrievers and Poodles commonly represented across all samples. Breeds overrepresented among dogs with adenomas included Labrador Retrievers, Golden Retrievers, Cocker Spaniels, Pugs, and Poodles, whereas epitheliomas occurred more frequently in Labrador Retrievers, Poodles, and mixed-breed dogs. Gross features revealed no significant differences between tumor types with respect to laterality (OD vs. OS) or eyelid location (upper vs. lower), indicating that tumor type does not influence anatomic distribution within the eyelid, despite a previous study documenting a higher prevalence of upper eyelid masses in dogs presenting to two Taiwanese veterinary referral hospitals (29). In the current study, epitheliomas demonstrated a significantly larger greatest tumor dimension when compared to adenomas, consistent with their more proliferative behavior. Interestingly, there was no significant correlation between tumor size and mitotic count for either MG tumor subtype. While tumor size, as assessed clinically or at grossing, and mitotic count often reflect related but distinct aspects of tumor biology, they are separated by multiple sources of bias; therefore, a tight statistical correlation is not necessarily expected. Specific reasons for the lack of correlation may include disparities between the duration and rate of tumor growth, regional heterogeneity in tumor proliferation, microenvironmental constraints on growth such as hypoxia or nutrient deprivation, or other clinical drivers of tumor size such as edema, chalazion, or cystic dilation, with resulting corneal trauma which may lead to earlier surgical indications.

Histologically, the two tumor subtypes demonstrated features consistent with their morphological distinctions. Adenomas were composed of well-differentiated meibocytes with abundant, foamy cytoplasm arranged in lobules. Epitheliomas exhibited a higher proportion of basaloid reserve cells and increased mitotic activity. Epitheliomas exhibited significantly higher mitotic counts than adenomas, reinforcing their more proliferative phenotype.

The current study was limited by conventional DNA detection methods, and the absence of detectable CPV-1 DNA does not definitively exclude papillomaviral infection. RNA-based detection methods such as RT-PCR and *in situ* hybridization (ISH) for CPV-1 mRNA may be more sensitive approaches to be considered in future studies. ISH approaches would also permit the direct visualization of transcriptionally active virus and provide morphologic evaluation of viral localization within infected neoplastic cells. Future research also aims to explore alternative etiologies for canine MG oncogenesis including genetic, epigenetic, and DNA damage mechanisms.

Here, we evaluated canine MG adenomas and epitheliomas for the presence of CPV-1 using DNA extracted from FFPE tumor samples. We demonstrated a paucity of viral amplification across all samples, and these data suggest that, unlike some human tumors, canine MG oncogenesis is not virally mediated.

## Data Availability

The original contributions presented in the study are included in the article/supplementary material, further inquiries can be directed to the corresponding author.

## Supplementary material

Supplementary Figure S1 Experimental design for evaluating canine Meibomian gland tumors for the presence of CPV-1.

Supplementary Figure S2 Correlation of greatest tumor dimension with mitotic count in adenomas **(A)** and **(B)** epitheliomas.

## References

[1] Roberts SM Severin GA Lavach JD. Prevalence and treatment of palpebral neoplasms in the dog: 200 cases (1975-1983). J Am Vet Med Assoc. (1986) 189:1355–9. doi: 10.2460/javma.1986.189.10.1355, 3793587

[2] Scott EM Miller PE. “Tumors of the eye and ocular adnexa”. In: DS Bruyette N Bex-field JD Chretin L Kidd S Kube C Langston , editors. Clinical Small Animal Internal Medicine. Hoboken, NJ, USA: John Wiley & Sons, Inc. (2020). p. 1261–9.

[3] Grahn B. Blepharitis and neoplasms of the canine eyelid margin and skin. Vet Clin Small Anim Pract. (2023) 53:455–71. doi: 10.1016/j.cvsm.2022.11.002, 36813395

[4] Peterson C Hicks JL De Marzo AM Campbell AA Eberhart CG Dubielzig RR . Upregulated MYC expression and p53 mutations may contribute to the oncogenesis of canine Meibomian gland carcinomas. Vet Pathol. (2023) 60:36541627:185–9. doi: 10.1177/03009858221143400 36541627

[5] Dubielzig RR Ketring K McLellan GJ Albert DM. "Chapter 7 - diseases of the eyelids and conjunctiva". In: Dubielzig RR Ketring K McLellan GJ Albert DM, editors. Veterinary Ocular Pathology. Edinburgh: W.B. Saunders (2010). p. 143–99.

[6] Tetzlaff MT Curry JL Ning J Sagiv O Kandl TL Peng B . Distinct biological types of ocular adnexal sebaceous carcinoma: HPV-driven and virus-negative tumors Arise through nonoverlapping molecular-genetic alterations. Clin Cancer Res. (2019) 25:1280–90. doi: 10.1158/1078-0432.CCR-18-1688, 30420449

[7] Moore RF Zhang XR Allison DB Rooper LM Campbell AA Eberhart CG. High-risk human papillomavirus and ZEB1 in ocular adnexal sebaceous carcinoma. J Cutan Pathol. (2021) 48:1027–33. doi: 10.1111/cup.13987, 33745190

[8] Saliba M Shaheen M El Hajj R Abbas FI Bashir S Sheikh UNN . Sebaceous neoplasms: prevalence of HPV infection and relation to immunohistochemical surrogate markers. Eur J Dermatol. (2021) 31:34001468:170–5. doi: 10.1684/ejd.2021.4008 34001468

[9] Peterson C Parikh RN Ahmad MT Campbell AA Daoud Y Mahoney N . Detection of human papillomavirus in squamous lesions of the conjunctiva using RNA and DNA in-situ hybridization. Int J Mol Sci. (2022) 23:7249. doi: 10.3390/ijms23137249, 35806252 PMC9266440

[10] Williams J Kostiuk M Biron VL. Molecular detection methods in HPV-related cancers. Front Oncol. (2022) 12:864820. doi: 10.3389/fonc.2022.864820, 35574396 PMC9092940

[11] Bartosik M Moranova L Izadi N Strmiskova J Sebuyoya R Holcakova J . Advanced technologies towards improved HPV diagnostics. J Med Virol. (2024) 96:e29409. doi: 10.1002/jmv.29409, 38293790

[12] Rector A Van Ranst M. Animal papillomaviruses. Virology. (2013) 445:213–23. doi: 10.1016/j.virol.2013.05.007, 23711385

[13] Munday JS Marshall S Thomson NA Kiupel M Heathcott RW French A. Multiple viral plaques with sebaceous differentiation associated with an unclassified papillomavirus type in a cat. N Z Vet J. (2017) 65:219–23. doi: 10.1080/00480169.2017.1313146, 28358996

[14] Munday J Grant K Orbell G Vaatstra B. Cutaneous plaques associated with a putative novel papillomavirus type in a horse. N Z Vet J. (2023) 71:100–5. doi: 10.1080/00480169.2022.2157347, 36484093

[15] Kuan I Tian K Grabosch S Sehn J Hoff J. HPV-associated vulvar carcinoma with sebaceous differentiation. Gynecol Oncol Rep. (2023) 50:101298. doi: 10.1016/j.gore.2023.101298, 37965381 PMC10641161

[16] Feng Y Wang K Zhou D Yuan Y Chen Y Wang J . Canine papillomavirus: status of diagnostic methods and vaccine innovations. Virol J. (2025) 22:128. doi: 10.1186/s12985-025-02753-3, 40307903 PMC12042422

[17] Chang C-Y Chen W-T Haga T Yamashita N Lee CF Tsuzuki M . The detection and Association of Canine Papillomavirus with benign and malignant skin lesions in dogs. Viruses. (2020) 12:170. doi: 10.3390/v12020170, 32028559 PMC7077320

[18] Brandes K Fritsche J Mueller N Koerschge B Dierig B Strebelow G . Detection of canine oral papillomavirus DNA in conjunctival epithelial hyperplastic lesions of three dogs. Vet Pathol. (2009) 46:34–8. doi: 10.1354/vp.46-1-34, 19112112

[19] Schaefer EAF Chu S Pearce JW Bryan JN Flesner BK. Papillomavirus DNA not detected in canine lobular orbital adenoma and normal conjunctival tissue. BMC Vet Res. (2019) 15:226. doi: 10.1186/s12917-019-1971-0, 31277650 PMC6612140

[20] Munday JS Knight CG Luff JA. Papillomaviral skin diseases of humans, dogs, cats and horses: a comparative review. Part 1: papillomavirus biology and hyperplastic lesions. Vet J. (2022) 288:105897. doi: 10.1016/j.tvjl.2022.105897, 36150643 PMC11494463

[21] Munday JS Knight CG Luff JA. Papillomaviral skin diseases of humans, dogs, cats and horses – a comparative review. Part 2: pre-neoplastic and neoplastic diseases. Vet J. (2022) 288:105898. doi: 10.1016/j.tvjl.2022.105898, 36152994 PMC11459685

[22] Wiggans KT Hoover CE Ehrhart EJ Wobeser BK Cohen LB Gionfriddo JR. Malignant transformation of a putative eyelid papilloma to squamous cell carcinoma in a dog. Vet Ophthalmol. (2013) 16:105–12. doi: 10.1111/j.1463-5224.2012.01062.x, 22882469

[23] de Villiers E-M Fauquet C Broker TR Bernard HU zur Hausen H. Classification of papillomaviruses. Virology. (2004) 324:17–27. doi: 10.1016/j.virol.2004.03.033, 15183049

[24] Griffin H Mudhar HS Rundle P Shiraz A Mahmood R Egawa N . Human papillomavirus type 16 causes a defined subset of conjunctival in situ squamous cell carcinomas. Mod Pathol. (2020) 33:74–90. doi: 10.1038/s41379-019-0350-5, 31485010 PMC6930848

[25] Cheng J Zens MS Duell E Perry AE Chapman MS Karagas MR. History of allergy and atopic dermatitis in relation to squamous cell and basal cell carcinoma of the skin. Cancer Epidemiol Biomarkers Prev. (2015) 24:749–54. doi: 10.1158/1055-9965.EPI-14-1243, 25670807 PMC4383698

[26] Jensen AO Svaerke C Körmendiné Farkas D Olesen AB Kragballe K Sørensen HT. Atopic dermatitis and risk of skin cancer: a Danish nationwide cohort study (1977-2006). Am J Clin Dermatol. (2012) 13:29–36. doi: 10.2165/11593280-000000000-00000, 22175302

[27] Arana A Wentworth CE Fernández-Vidaurre C Schlienger RG Conde E Arellano FM. Incidence of cancer in the general population and in patients with or without atopic dermatitis in the U.K. Br J Dermatol. (2010) 163:1036–43. doi: 10.1111/j.1365-2133.2010.09887.x, 20545690

[28] Beckwith-Cohen B Teixeira LBC Ramos-Vara JA Dubielzig RR. Squamous Papillomas of the conjunctiva in dogs: a condition not associated with papillomavirus infection. Vet Pathol. (2015) 52:676–80. doi: 10.1177/0300985814556185, 25352202

[29] Wang S-L Dawson C Wei L-N Lin C-T. The investigation of histopathology and locations of excised eyelid masses in dogs. Vet Rec Open. (2019) 6:e000344. doi: 10.1136/vetreco-2019-000344, 31897299 PMC6924796

