# Supplementary figures and images for: Papillomavirus is not detected in benign neoplasms of the canine Meibomian gland despite evidence of HPV-mediated tumorigenesis in the human Meibomian gland
Source: Front Vet Sci. 2026 Apr 9;13:1812954. doi: 10.3389/fvets.2026.1812954 (PMC13102608; doi:10.3389/fvets.2026.1812954)

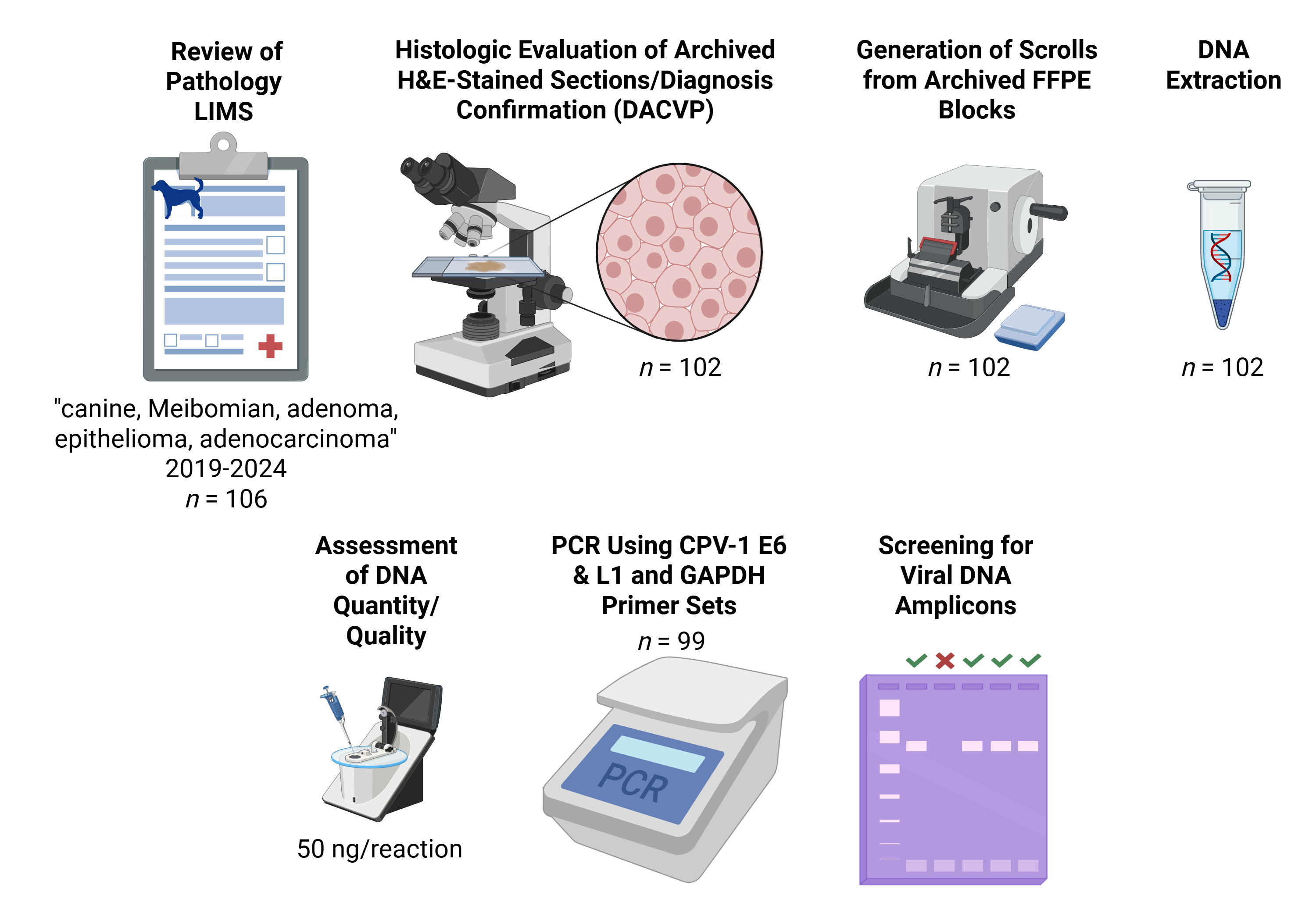

Supplement: Supplementary Figure S1 — Experimental design for evaluating canine Meibomian gland tumors for the presence of CPV-1. [file Image_1.TIF]

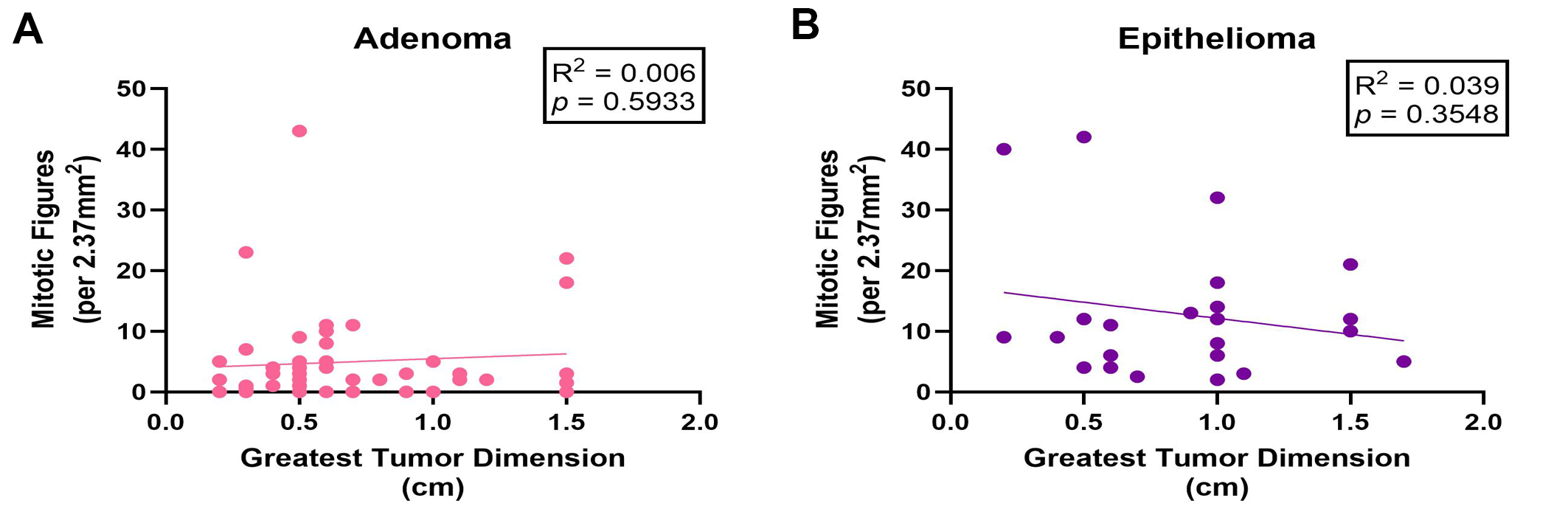

Supplement: Supplementary Figure S2 — Correlation of greatest tumor dimension with mitotic count in adenomas (A) and (B) epitheliomas. [file Image_2.TIF]
